# Intracranial Atherosclerosis Burden and Stroke Recurrence for Symptomatic Intracranial Artery Stenosis (sICAS)

**DOI:** 10.14336/AD.2018.0301

**Published:** 2018-12-04

**Authors:** Ping Sun, Liping Liu, Yuesong Pan, Xianwei Wang, Donghua Mi, Yuehua Pu, Xin Liu, Wanying Duan, Hongyi Yan, Chunxue Wang, Xingquan Zhao, Yilong Wang, Yongjun Wang, on behalf of CICAS investigators

**Affiliations:** ^1^Department of Neurology, Beijing Tiantan Hospital, Capital Medical University, Beijing, China.; ^2^China National Clinical Research Center for Neurological Diseases, Beijing, China.; ^3^Center of Stroke, Beijing Institute for Brain Disorders, Beijing, China.; ^4^Beijing Key Laboratory of Translational Medicine for Cerebrovascular Disease, Beijing, China.; ^5^Department of Epidemiology and Health Statistics, School of Public Health, Capital Medical University, Beijing, China.; ^6^Beijing Municipal Key Laboratory of Clinical Epidemiology, Beijing, China.; ^7^The Second People's Hospital of Guiyang, Guizhou, China.; ^8^Department of Medicine and Therapeutics, Prince of Wales Hospital, Chinese University of Hong Kong, Shatin Hong Kong SAR.

**Keywords:** Intracranial Atherosclerosis Burden, ICAS, recurrence

## Abstract

Intracranial atherosclerosis burden is an arising key index for the risk and prognosis for Intracranial Atherosclerosis Stenosis (ICAS). The present study estimated one-year prognosis for patients of symptomatic ICAS with different degrees of intracranial atherosclerosis burden (ICASB) and identified whether the category of multiple and single acute infarction was associated with atherosclerosis burden. A total of 2864 consecutive patients, from 22 hospitals across China, who experienced an acute cerebral ischemia <7 days after onset of symptoms were evaluated. All patients underwent magnetic resonance angiography, and the degree of intracranial stenosis with the ICASB was calculated. The patients were categorized into three groups according to ICASB grading: <4, 4-5 and >5scores. Multivariate Cox proportional hazards regression models were used to estimate the impact of the hazard ratios(HR) of the putative determinants of recurrent stroke in one year. In the groups with ICASB 4-5 and ICASB >5scores recurrent stroke were significantly higher than the other (*P*<0.0001). On multivariate logistic analysis, ICASB (4-5) indicated more stroke recurrence at 12 months (adjusted hazard ratio, 1.96; 95% confidence interval, 1.08-3.56; *P*=0.027), compared to the ICASB<4scores and >5 groups (*P*<0.001). Moreover, proportion of single and multiple infarction lesions differs with different ICASB. Multiple lesions were related with higher of ICASB(P<0.001). Intracranial atherosclerosis burden was associated with recurrent stroke at 12 months. Multiple infarction lesions were associated with higher ICASB score which indicate higher risk of recurrent.

Intracranial atherosclerotic stenosis (ICAS) is a main etiology of ischemic stroke, especially in Asian patients [1, 2]. Previous studies [3, 4] had shown that multiple location of ICAS were independent predictors of unfavorable outcome at 6 months. In addition, the patients exhibiting recent symptoms of intracranial stenosis face a maximal risk of ≥70% of subsequent stroke in the territory of the stenotic artery. The Chinese Intracranial Atherosclerosis (CICAS) study [5] reported that severe stenosis and multiple stenosis are risk factors for recurrent stroke. Moreover, intracranial athero-sclerosis burden (ICASB) is regarded as the index more reflective of pathology for ICAS patients. Although the differ outcome of patients with the different degrees of stenosis were observed, but the recruitment in this group were moderate or severe stenosis of >50%, who could be considered as a group of high recurrent rate.

From the report of the TIAregistry [6], indicated that patients with multiple acute infarction lesions had more than double of stroke recurrence compared to those with single acute infarction. This study aimed to examine the recurrent of ICAS among patients presenting acute ischemic stroke, in order to identify the prognostic value of atherosclerosis burden and related imaging category of lesions.

## MATERILAS AND METHODS

### Patients and Workups

Chinese Intracranial Atherosclerosis (CICAS) is a prospective, multicenter, hospital-based study, encompassing twenty-two general hospitals. The study had been approved by the Institutional Review Board at the participating hospitals, and informed consent was obtained from each participant prior to the study. Clinical and imaging data were prospectively collected from patients with ischemic stroke (IS) or transient ischemic attack. Patients who presented the onset of symptoms in <7 days were enrolled in the study and were aged between 18 and 80 years. Patients who were clinically unstable or required close monitoring or were disabled before admission (modified Rankin scale (mRS) score >2) and were physically or subjectively unable to comply with the magnetic resonance (MR) examination were excluded. Patients with cardio-embolic risk factors (atrial fibrillation, valvar heart disease, postcardiac valve replacement) and undetermined causes were also excluded.

### Clinical Information Assessment

Clinical information included age, sex, and vascular risk factors, such as hypertension (defined by a history of hypertension, or diagnosed at discharge), diabetes mellitus (defined by a history of diabetes mellitus or diagnosis at discharge), hyperlipidemia (defined as low-density lipoprotein cholesterol more than or equal to 2.6 mmol/L at admission or a history of hyperlipidemia or receiving lipid-lowering treatment or diagnosis at discharge), history of IS or intracerebral hemorrhage, heart disease (defined as a history of myocardial infarction, angina pectoris, and congestive heart failure). National Institutes of Health Stroke Scale (NIHSS) score at admission and discharge, and mRS score at discharge and 1 year after stroke onset were evaluated. Smoking history, current or previous smokers (continuously smoking ≥1 cigarette a day for 6 months), and a history of heavy alcohol use (drinking >2 U/day on average for men or >1U/day on average for women) was also collected. In addition, we recorded the using of antithrombotic therapy within 48 hours of admission, at discharge and 1 year after stroke onset.

### Image Interpretation

All patients underwent conventional MRI on a 3.0Tor 1.5T MR scanner. The imaging sequences included a 3-dimensional time-of-flight MRA (repetition time/echo time (TR/TE), 20-25/3.3-3.9 ms; flip angle, 15-20°; slice thickness, 0.65-1.00 mm), T2/T1-weighted imaging (repetition time, 4500 ms; echo time, 84 ms; repetition time, 1200 ms; echo time, 11 ms), fluid-attenuated inversion recovery sequences (repetition time, 7000 ms; echo time, 94 ms), and diffusion-weighted imaging (repetition time, 3000 ms; echo time, 75 ms). Two neurologists interpreted the MRI images. In the event of disagreement, a third reader was invited to resolve the issue and reach a consensus.

### The Definition of ICASB

The degree of intracranial stenosis on MRA was calculated based on the method from the WASID study [7]. The degree of ICAS was measured in each patient. The atherosclerotic lesions of intracranial vessels on magnetic resonance angiography (MRA) were visually graded: 0, no stenosis; 1, stenosis <50%; 2, stenosis 50-99%; and 3, occlusion. The assessment of the ICAS location included MCA, anterior and posterior cerebral arteries, basal artery (BA), and intracranial portions of the internal carotid and vertebral arteries. The sum of the involved intracranial vessels was defined as the ICASB according to prior study in the subgroups [8]. The patients were categorized into three groups according to ICASB: (1) <4 scores; (2) 4-5 scores; (3) >5scores.

We estimated the risk of stroke in subgroups of patients categorized according to the time from diffusion-weighted imaging to evaluation by a stroke specialist and the number of acute (new) infarction-related lesions (single infarction *vs*. > multiple infarctions).

### Outcome Measures

The primary outcome was progressive deterioration or recurrence of IS in 1 year. The definition of progressive deterioration of IS had worsened by ≥4 points of the initial NIHSS score from the index stroke, a new focal neurological deficit of vascular origin lasting for >24 h was defined as stroke recurrence. Patients or their authorized proxies were contacted over the telephone at 3, 6, and 12 months after discharge, in order to monitor whether patients experienced new symptoms or were hospitalized again because of another stroke; these data were collected by trained research personnel at Beijing Tian Tan hospital. In addition, the recurrence or progressive deterioration of IS were verified based on the NIHSS score and the presence of new neurological deficits documented in the medical records. The patients’ medical documents were reviewed by an experienced stroke neurologist to ensure a reliable diagnosis of recurrence or progressive deterioration of IS. In case of missing any personal information or dead without hospitalization, a stroke neurologist or investigators made the decision based on the medical record. Any death was verified by examining the hospital medical records or local citizen registry. At discharge and 1 year after stroke onset, the daily activities were assessed by mRS [9]. The functional dependence was defined as mRS>2. The stroke severity at admission and discharge was measured by the NIHSS [10] based on the stroke onset.

**Table 1:** Baseline Characteristics of the Participants.

Characteristics	Total, N (%) 2864	ICASB (<4) (n=2401)	ICASB (4-5) (n=214)	ICASB (>5) (n=150)	*p*-value
Demographic					
Male, n (%)	1944(67.9)	1706(68.5)	138(64.4)	100(62.8)	0.19
Age (mean±SD), year	61.91±11.2	61.71±11.3	63.24±10.4	63.2±11.0	0.06
Vascular risk factors					
Diabetes mellitus, n (%)	699 (24.7)	569 (23.0)	77 (36.7)	53 (34.6)	<0.0001
Hypertension, n (%)	1898 (66.8)	1612 (65.2)	150 (75.0)	127(81.4)	<0.0001
Hyperlipidemia, n (%)	450 (18.2)	378 (17.5)	37(21.4)	35(25.7)	0.0295
Family history of stroke, n (%)	296 (10.5)	236 (9.6)	34(15.1)	26(16.3)	0.0006
previous and current smoker, n (%)	1296 (45.8)	1138 (46.2)	91 (43.3)	67 (42.9)	0.55
History of IS, n (%)	710 (24.8)	575 (23.1)	62 (29.0)	73(45.9)	<0.0001
History of hemorrhage stroke, n (%)	54 (1.1)	46 (1.9)	3 (1.4)	25(3.1)	0.44
Heart disease, n (%)	228 (7.9)	186 (7.7)	26(12.2)	16 (10.1)	0.03
Peripheral vascular disease, n (%)	21 (0.7)	16 (0.6)	4 (1.9)	1 (0.6)	0.13
Heavy drinker, n (%)	407 (14.2)	355 (14.3)	32 (14.9)	20 (12.6)	0.79
Severity of ICAS					<0.001
None	1551 (54.2)	1551 (62.3)	0	0	
<50%	393 (13.7)	390 (15.7)	3 (1.4)	0	
50-99%	505 (17.6)	338 (13.6)	123 (57.5)	44 (27.7)	
100%	415 (14.5)	212 (8.5)	88 (41.1)	115 (72.3)	
Performance measures					
Antihypertension, n (%)	1392 (48.7)	1191 (47.9)	109 (51.6)	92(57.9)	0.04
Early antithrombotic, n (%)	2494 (87.0)	2186 (87.7)	177 (82.7)	32 (20.1)	0.0065
Statins, n (%)	2170 (75.8)	1860 (74.7)	180 (84.1)	130(81.8)	0.0016
NIHSS score at admission(IQR)	4(1-7)	3 (1-6)	4 (2-8)	5 (2-10)	0.0001
Pre-admission MRS<2 scores, n (%)	2857 (100)	2484 (87.0)	213 (7.5)	159 (5.6)	0.02

ICASB: intracranial atherosclerosis burden; NIHSS: National Institutes of Health Stroke Scale; mRS: modified Rankin Scale; IS: ischemic stroke IQR: interquartile range.

**Table 2:** Univariate analysis of outcome with recurrent stroke at 12 months.

Characteristics	Total (n=2864)	Recurrent stroke (n=100)	No-recurrent stroke (n=2764)	p-value
Male sex	1944 (67.9)	84 (64.6)	1860 (68.0)	0.41
Age (mean±SD), year	61.91±11.2	65.47±10.8	61.74±11.2	0.0002
Diabetes mellitus, n (%)	699 (24.7)	42 (32.8)	657 (24.3)	0.03
Hypertension, n (%)	1898 (66.8)	97 (74.6)	1801 (66.4)	0.05
Hyperlipidemia, n (%)	450 (18.2)	17 (15.6)	433 (18.4)	0.46
Familyhistory of stroke, n (%)	296 (10.5)	21 (16.8)	275 (10.2)	0.02
Previousandcurrent smoking, n (%)	1296 (45.8)	45 (34.9)	1251 (46.3)	0.01
Heavy drinker, n (%)	407 (14.2)	13 (10.0)	394(14.4)	0.16
History of IS, n (%)	710 (24.8)	50 (38.5)	660 (24.1)	0.0001
History of hemorrhage stroke, n (%)	54 (1.9)	1 (0.8)	53 (1.9)	0.34
History of heart disease, n (%)	228 (8.0)	19(14.6)	209 (7.6)	0.004
Peripheral vascular disease, n (%)	21 (0.7)	1 (0.8)	20 (0.7)	0.96
Body mass index	24.58±3.2	24.62±2.4	24.57±3.2	0.87
SBP at admission, mmHg	150.7±23.4	155.26±22.9	150.49±23.4	0.03
DBP at admission, mmHg	87.73±13.4	89.72±12.6	87.62±13.4	0.09
NIHSS score at admission	4 (1-7)	5 (2-10)	3 (1-7)	<0.0001
Pre-admission mRS<2 scores, n (%)	2857 (100)	128(4.5)	2729(95.5)	0.03
Performance Measures				
Early antithrombotic therapy, n (%)	367 (12.8)	25 (19.4)	3425(12.5)	0.022
Statins, n (%)	2170(75.8)	93 (71.5)	2077 (76.0)	0.25
Antihypertension, n (%)	1392 (48.7)	67(51.9)	1341 (48.6))	0.45
Severity of ICAS				0.0006
None	1551(54.2)	51(39.2)	1500(54.9)	
<50%	393(13.7)	20(15.4)	373(13.6)	
50-99%	505(17.6)	26(20.0)	479(17.5)	
100%	415(14.5)	33(25.4)	382(14.0)	
ICASB				<0.0001
ICASB (<4)	2491 (87.0)	97 (74.6)	2394 (87.6)	
ICASB (4-5)	214 (7.5)	22 (16.9)	192 (7.0)	
ICASB (>5)	159(1.9)	11 (8.5)	148(5.4)	
Category of infarction				0.038
Single infarction	1864 (86.5)	87 (79.8)	1777 (86.8)	
Multiple infarctions	292 (13.5)	22(20.2)	270 (13.2)	
mRS at 12 months (3-6)	507(19.0)	70 (56.0)	437(17.1)	<0.0001

DBP, diastolic BP; SBP, systolic BP; ICASB, intracranial atherosclerosis burden; NIHSS, National Institutes of Health Stroke Scale. * Continuous variables are expressed as mean±SD; other values are expressed as n (%).

### Statistical Methods

Continuous variables with non-normal distribution were summarized as median (interquartile range). The categorical variables such as male sex and vascular risk factors were presented as absolute numbers and percentages. The comparison of continuous variables utilized independent samples t-test or Wilcoxon test, whereas the χ^2^ test or Fisher’s exact test was used for the comparison of categorical variables. The adjusted differences in NIHSS score at admission and hospital stay for patients’ single and multiple infarctions were analyzed by the general linear model. The univariate and multivariate Cox proportional hazards regression models were used to estimate the impact of the hazard ratios (HR) of the putative determinants of recurrent stroke. All probability values were 2-tailed; *P*<0.05 was considered statistically significant. All analyses were performed using SAS version 9.1 (SAS Institute, Cary, NC, USA).

## RESULTS

The cohort consisted of 2864 consecutive patients from October 2007 to June 2009. The follow-up was continued for 12 months; 176 (6.2%) patients were lost to follow-up.

Baseline of the clinical features of the participants were summarized in Table 1. The mean age at admission for the index stroke was 61.9±11.2 years, and 67.9% of the patients were male. Hypertension (66.8%), hyperlipidemia (18.2%), diabetes mellitus (24.7%), and history of cerebral ischemia (24.8%) were the most common vascular risk factors. During the hospital stay, the common treatments included early antithrombotic therapy (87.0%), statins (75.8%), and antihypertensive drugs (48.7%). According to the three groups of ICAS burden, patients with high ICAS burden were likely to be older and had risk factors such as diabetes mellitus, hypertension, family history of stroke, and history of cerebral ischemia. The admission NIHSS score was high in the groups of high ICASB (*P*<0.0001). The severity of intracranial artery stenosis differs associating with burden degrees, indicating in those with >4 scores obtained more proportion over 50% ICAS (*P*<0.0001). Moreover, mRS score at before admission was low in the groups of higher ICASB (*P*=0.02).

**Table 3:** Multivariate analysis for predictors of recurrent stroke at 12 months.

Predictors	HR (95%Cl)	*p*-value
ICASB (4-5)	1.96 (1.08-3.56)	0.027
Family history of stroke	2.03 (1.21-3.41)	0.0072
History of ((IS)	1.76 (1.14-2.73)	0.0096
History of heart history	1.40 (0.74-2.67)	0.298
NIHSS score at admission	1.05 (1.00-1.09)	0.028

CI indicates confidence interval; HR, hazard ratio; ICASB, intracranial atherosclerosis burden; NIHSS, National Institutes of Health Stroke Scale; IS, ischemic stroke.

Univariate analysis showed that several factors were associated with recurrent stroke (Table 2). The family history of stroke, history of cerebral ischemia, heart disease, previous or current smoking and high NIHSS at admission, the moderate or severe artery stenosis (>50%) were correlated to recurrent stroke (*P*<0.05). Moreover, patients with ICASB>4 scores showed a significantly higher rate of recurrent stroke (*P*<0.0001). For the lesion category, group with multiple infarctions were more likely associated with the risk of recurrence than the single infarction group (*P*=0.038). In multivariate analysis, ICASB (4-5) (HR: 1.96; 95% CI: 1.08-3.56; *P*=0.027), family history of stroke (HR: 2.03; 95% CI: 1.21-3.41; *P*=0.0072), history of cerebral ischemia (HR: 1.76; 95% CI: 1.14-2.73; *P*=0.0096) were correlated to the recurrence of stroke. Besides, NIHSS score at admission, family history of stroke and history of ischemic stroke were independent predictors for recurrent stroke as well (Table 3).

**Figure 1.**
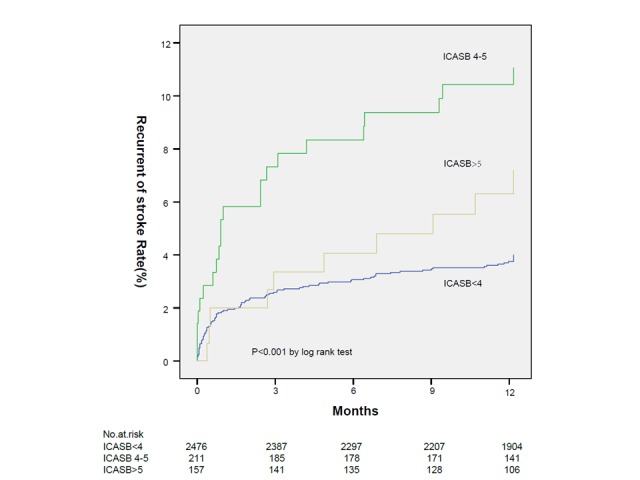
Kaplan-Meier curves for stroke recurrence from the time of the qualifying event up to 1 year in the three groups. ICASB, intracranial atherosclerosis burden.

**Figure 2.**
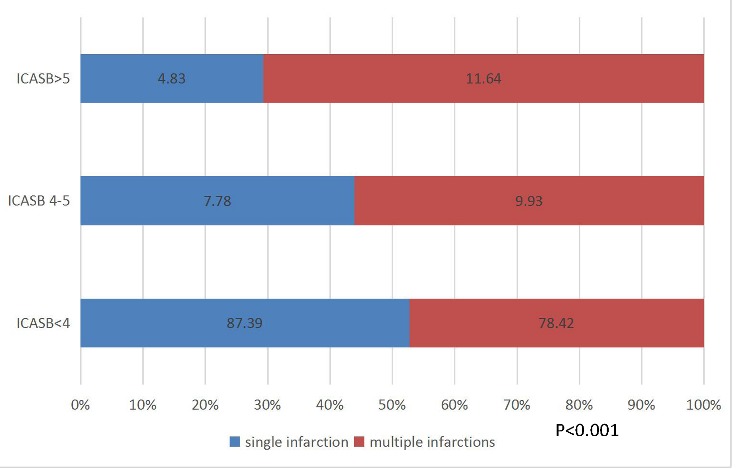
Proportion of infarction lesions in different ICSB group. Multiple infarctions were seen more than single infarction in group ICASB >4 scores (P<0.001). P-value was obtained by Chi-square test to assess the association of intracranial atherosclerosis burden (ICASB) in single infarction and multiple infarctions.

Kaplan-Meier analysis showed that the ICASB (4-5) groups was associated with recurrent stroke at12 months as compared to the ICASB <4 scores group and >5 scores (Fig. 1). The proportion of single and multiple infarction lesions differs with different ICASB, higher percentage of multiple lesions in the groups of higher burden >4 scores (*P*<0.001) (Fig. 2).

## DISCUSSION

We evaluated outcomes of the patients with the different ICASB scores. ICASB can be assessed by different methods. One of prior study [3] calculated ICASB by evaluating intracranial only, concurrent extra-intracranial lesions, extracranial only, recent study [11] calculated ICASB according to the numbers of artery stenosis which relative to clinical outcomes. In our study, most of traditional vascular risk factors such as diabetes mellitus, hypertension, family history of stroke, and history of cerebral ischemia were associated with higher ICASB (P<0.0001), and NIHSS score at admission was higher in the groups of higher ICASB (P<0.0001), and the ratio of mRS score <2 at pre-admission was lower in the groups of higher ICASB (P=0.02). In the Warfarin-Aspirin Systematic Intracrinal Disease (WASID) study [12], this variety of symptomatic intracranial stenosis was independently related to a higher risk of subsequent stroke in the territory of the stenotic artery. The risk of recurrent stroke in the territory of the symptomatic stenotic artery was as high as 23% during the first year in patients with stenosis ≥70%. The current study showed that patients with ICASB (>4 scores) had a significantly high risk of recurrent stroke, and ICASB (>4 scores), family history of stroke, history of cerebral ischemia and NIHSS score at admission were independent predictors of recurrent stroke. Moreover, the proportion of single and multiple infarction lesions differs with different ICASB, the higher of burden, more of multiple lesions.

The current study showed that the ICASB (4-5scores) group were more associated with recurrent stroke in 12 months compared to the ICASB <4 scores and >5 group showing to a large number of 50-99% stenosis arteries in the group, and also result from little samples in >5 group. Thus, single infarction and multiple infarctions might have different ICASB, the higher portion of multiple infarction lesions were found in group with higher ICASB scores, this more relative to most of clinic interpretations of multiple lesion, showing high risk of embolism and recurrence, and this observation was in agreement with the previous study [6].

Moreover, our study show high stroke recurrence at 12 months among patients with severe stenosis, which were indicated the same results in the most recently RCTs of WASID [12] and SAMPRIS [13]. ICAS of <50% and 50-69% luminal stenosis is usually regarded as nonsignificant or defined mild or moderate lesions respectively [12, 14], however, ICASB (4-5) group was highly related to recurrent stroke in our study, and there were more patients with mild stenosis in this groups. Therefore, recurrent risks in patients with mild stenosis need to be fully appreciated in future studies, so that high-risk patients would not be missed because of artificially graded severity of ICAS by the degree of luminal stenosis [13]. And then, different stroke mechanisms accompany with ICAS, and different aetiological mechanisms has significant clinical implications [15]. Recently study show Interleukin-33 (IL-33) and levels of fibrinogen after anticoagulation were related to ischemic stroke [16,17]. so the evaluation of intracranial atherosclerotic disease should be reconsidered from grading of stenosis to hemodynamic and emboligenic lesion characterization because of a large mountain of evidence about collaterals, hemodynamic impact, and other factors in determining subsequent stroke risk in patients with symptomatic ICAS [18-20], so we should optimally identify those who are truly at high risk.

The study had several limitations. First, the intracranial large artery was evaluated by MRA. Although it was flow-sensitive and not as accurate as DSA, it is difficult to differ vascular stenosis from 50-70% to 70-99%. However, MRA was noninvasive and easily accessible as compared to DSA [21]. Second, we could not accurately exclude a reanalyzing embolus from an *in situ* stenosis although the patients with presumed cardio-embolism were excluded. Third, intracranial vasculitis or primary agilities of the central nervous system (PACNS) were not excluded specifically. Fourth, we did not record the changes in medications during the 12months follow-up period.

In conclusion, we found that ICASB was associated with recurrent stroke. Multiple infarctions had higher ICASB scores for symptomatic intracranial artery stenosis patients. Except severity of stenosis, this might be providing an additional assessment method for mechanism and prognosis of sICAS patients.
